# Supplementary material for: How do and could clinical guidelines support patient-centred care for women: Content analysis of guidelines
Source: PLoS One. 2019 Nov 8;14(11):e0224507. doi: 10.1371/journal.pone.0224507 (PMC6839851; doi:10.1371/journal.pone.0224507)
Supplement: S1 Table — (DOCX) [file pone.0224507.s002.docx]

**Supplementary File 1. MEDLINE and EMBASE Search Strategy**

**Mental Health (Depression)**

MEDLINE

--------------------------------------------------------------------------------

1 Depression/ or Depressive Disorder/ or Depressive Disorder, Major/ (186961)

2 depression.mp. (317868)

3 depress*.mp. (441037)

4 1 or 2 or 3 (441037)

5 1 and 2 and 3 (159074)

6 GUIDELINE ADHERENCE/ or GUIDELINE/ or PRACTICE GUIDELINE/ (57979)

7 guideline*.mp. (347258)

8 6 or 7 (347258)

9 6 and 7 (57979)

10 4 and 8 (6328)

11 limit 10 to yr="2010 -Current" (3130)

12 limit 10 to (english language and yr="2010 -Current") (2798)

13 limit 11 to (english language and yr="2010 -Current" and english and practice guideline) (96)

EMBASE

--------------------------------------------------------------------------------

1 postnatal depression/ or minor depression/ or depression/ or perinatal depression/ or major depression/ or depression.mp. or antenatal depression/ (594990)

2 depress*.mp. (701371)

3 1 or 2 (701371)

4 guideline.mp. or practice guideline/ (379138)

5 guideline*.mp. (622855)

6 4 or 5 (622855)

7 3 and 6 (17448)

8 limit 7 to (english language and yr="2010 -Current") (9904)

9 limit 8 to full text (2355)

**Cardiac Rehabilitation**

MEDLINE

--------------------------------------------------------------------------------

1 cardiovascular disease.mp. or Cardiovascular Diseases/ (184246)

2 cardiac rehabilitation.mp. or Cardiac Rehabilitation/ (5338)

3 1 or 2 (188406)

4 1 and 2 (1178)

5 GUIDELINE ADHERENCE/ or guideline.mp. or GUIDELINE/ or PRACTICE GUIDELINE/ (85725)

6 guideline*.mp. (347258)

7 5 or 6 (347258)

8 4 and 7 (168)

9 limit 8 to (yr="2010 -Current" and english and practice guideline) (16)

EMBASE

--------------------------------------------------------------------------------

1 cardiovascular disease.mp. or cardiovascular disease/ (315754)

2 cardiac rehabilitation.mp. or heart rehabilitation/ (11523)

3 1 and 2 (1891)

4 guideline.mp. or practice guideline/ (379387)

5 guideline*.mp. (623212)

6 4 or 5 (623212)

7 3 and 6 (331)

8 limit 7 to (english language and yr="2010 -Current") (200)
