# Supplementary material for: How do and could clinical guidelines support patient-centred care for women: Content analysis of guidelines
Source: PLoS One. 2019 Nov 8;14(11):e0224507. doi: 10.1371/journal.pone.0224507 (PMC6839851; doi:10.1371/journal.pone.0224507)
Supplement: S2 Table — (DOCX) [file pone.0224507.s003.docx]

**Supplementary File 2. PCC framework domains and subdomains [7]**

| Fostering relationship | Exchanging information | Managing patient emotions | Addressing uncertainty | Making decisions | Enabling patient self-management |
| --- | --- | --- | --- | --- | --- |
| - Discuss roles and responsibilities - Honesty, openness, disclosure - Trust in clinician’s technical competence, skills and knowledge - Expression of caring and commitment - Building rapport and connection | - Exploring knowledge, beliefs and information needs and preferences - Sharing information - Providing informational resources; helping patients/family members evaluate and utilize resources - Facilitating assimilation, understanding and recall of information | - Expression of emotions - Exploring and identifying emotions - Assessing depression, anxiety or psychological distress - Validation of emotions - Expression of empathy, sympathy and reassurance - Providing tangible help in dealing with emotions | - Constructing and defining uncertainty - Assessing and understanding uncertainty - Using emotion-focused management strategies - Using problem-focused management strategies | - Communicating about decisional needs, decision support and decision process - Preparation for the decision and deliberation - Making and implementing a choice and action plan - Assessing decision quality and reflecting on choice | - Learning and assessing - Sharing and advising - Prioritizing and planning - Preparing, implementing and assisting - Arranging and following-up |
