# Supplementary material for: How do and could clinical guidelines support patient-centred care for women: Content analysis of guidelines
Source: PLoS One. 2019 Nov 8;14(11):e0224507. doi: 10.1371/journal.pone.0224507 (PMC6839851; doi:10.1371/journal.pone.0224507)
Supplement: S3 Table — (DOCX) [file pone.0224507.s004.docx]

**Supplementary File 3. Data extracted from included guidelines**

**Depression**

| Developer  (year, country) [ref] | Development instrument or system | Fostering Relationship | Exchanging Info | Responding to Emotions | Managing Uncertainty | Making Decisions | Enabling Self-Management | Other PCC Elements Mentioned | General/Any Mention | Women’s Health |
| --- | --- | --- | --- | --- | --- | --- | --- | --- | --- | --- |
| Canadian Partnership Against Cancer (CPAC) and the Canadian Association of Psychosocial Oncology (CAPO). CAPO is the steward of this guideline.  ,2015, Canada [32] | AGREE, GRADE | N/A | Patients should be counseled on the specific symptoms of depression or when distress is severe enough to warrant a call to the physician or nurse, or psychosocial oncology expert. Consider use of patient handouts. | All health care providers should routinely screen for the presence of emotional distress from the point of diagnosis onwards. Concerns such as risk of harm to self/others, severe depression or agitation may require an urgent referral to psychiatrist, psychologist, physician or equivalently trained professional. Psychosocial health services must focus on meeting the individual’s physical, social, emotional, nutritional, informational, psychological, spiritual, and practical needs. | Patients with distress may express fear, worry, uncertainty about the illness and future, sadness, anger, poor sleep, poor appetite, poor concentration, pre-occupation with thoughts of illness and death, and concerns about roles and relationships. Such distress may require psychosocial and supportive care or specific medications to manage symptoms. | N/A | N/A | N/A | N/A | N/A |
| The Centre of Perinatal Excellence (COPE), 2017, Australia [31] | 2016 National Health and Medical Research Council Standards for Guidelines, AGREE | Establishing and maintaining a therapeutic relationship between the health professional and the woman and her significant other(s)  is essential to providing psychosocial care in the perinatal period. Continuity of carer is likely to improve or facilitate the therapeutic  relationship. Important aspects include the development of trust, confidence, mutuality, active listening and empowerment. Clinicians should build rapport, maintain a non-judgemental attitude and address any feelings of stigma. | High quality information for women, their families is crucial. be given easily understandable explanations in her first language of the details of her specific health problem, any proposed  treatments or procedures and the results of any tests performed. Consistency of information,  especially if this is provided by different professionals, is very important. | Regular enquiry about emotional wellbeing provides a woman with opportunities for discussion about how she is managing and  allows health professionals to determine whether repeat depression screening or other assessments are indicated. Provides options for online emotional support apps and websites. | Acknowledge and describe, if possible, the uncertainty around any estimate of risk, harm or benefit | Care should involve collaborative decision-making with the woman and her significant other(s) if the woman agrees,  which includes full discussion of the potential risks and benefits of any treatments offered. | Identify appropriate health professionals available to provide follow-up care and to assist if  there are concerns for the safety of the woman | N/A | N/A | Many women live in poverty, subsisting on pensions or low-income occupations, restricted by  under-employment and experiencing poor health outcomes. Gender inequalities persist, with women economically  less secure, maintaining the primary carer role, and subject to violence (including physical and sexual assault, as well as emotional,  psychological and financial abuse). Aboriginal women, migrant women and lesbian, gay or transgender women face discrimination. |
| American College of Physicians, 2016, United States [37] | 2010 American College of Physicians Development of Clinical Practice Guidelines and Guidance Statements | N/A | N/A | Discusses various treatments for depression throughout document (ex. CBT, interpersonal therapy, psychodynamic therapies (pg. 353-354). | N/A | Patients and physicians should discussion adverse event profiles of anti-depressants before making a decision (pg. 356). | N/A | N/A | N/A | N/A |
| Canadian Network for Mood and Anxiety Treatments, 2016, Canada [36] | GRADE | N/A | N/A | Refers to DSM-5 for assessing for psychiatric disorders such as depression (pg. 554) and identifies methods of helping dealing with symptoms of depression via various treatments throughout the document (ex. Patient factors that can help select an antidepressant on pg. 543). | N/A | Selection of an anti-depressant should be based on the patients individualized needs, preferences and perceptions and also include physician expertise (pg. 542). | N/A | N/A | Refers to patient perceptions, preferences, and individualized needs during antidepressant selection (pg. 542). | N/A |
| Institute for Clinical Systems Improvement, 2016, United States [40] | GRADE | Suggests that it is important to establish a therapeutic alliance between patient and clinician regardless of treatment strategy and despite time constraints (pg. 4) | Recommends that clinicians educate and engage patient about treatment options (pg. 4). | Recommends use of DSM-5 for diagnosing depressive disorders (pg. 2) and recommends various treatments to help patient manage depressive symptoms throughout the guideline. | N/A | Recommends that shared decision making be used when deciding between medication and therapy as treatment (pg. 3, 4) | Strongly recommends that clinicians establish and maintain follow up with patients (pg. 5). | N/A | Refers to patient-centered care, which considers patient preferences in implementation of the guideline (pg. 18) | Strongly recommends that clinicians monitor and screen for depression in pregnant and postpartum women because it has been associated with negative pregnancy outcomes (pg. 5) |
| National Institute for Health and Care Excellence, 2016, United Kingdom [44] | National Institute for Health and Care Excellence Developing NICE Guidelines | Decisions should be made in partnership between patient and clinician and that good communication between the two parties is essential (pg. 8). Suggests that clinicians should build trusting relationships, and provide an open, non-judgemental and engaging environment (pg. 12) | Information should be:   - Tailored to patients’ needs (pg. 8) - Culturally appropriate (pg. 8) - Shared with families and carers where appropriate (pg. 8, 14) - Address common misconceptions (pg. 10) - Confidential and private (pg. 12) - Appropriate to patients’ level of understanding regarding disease and treatment (pg. 13) - Avoid medical jargon (pg. 13) - Available in written and/or audio format in patient's language or clinicians should be willing to work with interpreters (pg. 13, 16, 17) - Discuss discontinuation symptoms if considering stopping or reducing medication (pg. 34) | Clinicians should provide support and encouragement to prevent relapse (pg. 10). Discussions should take place in an environment fostering hope and optimism (pg. 12) | Clinicians should discuss uncertainty of the effectiveness of counselling and psychodynamic psychotherapy for treatment of depression (pg. 23). Evidence for use of light therapy indicates its efficacy is uncertain (pg. 28) | Patients should have the opportunity to make informed decisions about their care and treatment (pg. 8). Families and carers should also be involved in decision making where appropriate (pg. 8). Patient preferences should be considered when deciding what treatment is right for them (pg. 33) | Patients should be informed about self-help groups, support groups and other resources (pg. 13). Regarding suicide, assess whether the patient has a support network and take steps to mitigate risk (pg. 15). | Stigma, discrimination, dignity and respect should be addressed (pg. 12) | Person-centred care should take into account patients' needs and preferences (pg. 8) | N/A |
| Registered Nurse's Association of Ontario, 2016, Canada [34] | Healthcare Improvement Scotland (SIGN) 50: A guideline developers handbook, AGREE, AMSTAR | Clinicians should be self-aware, educated and reflect on their attitude, behaviour, communication style, and language during interactions with patient (pg. 31)  Clinician-patient relationship should involve (pg. 37):   - Establishing trust and rapport - Empathy - Self-awareness - Self-knowledge - Effective communication skills | Clinicians should:   - Note what quality of life means to patient since it’s subjective (pg. 32) - Communicate respect and care should verbally and non-verbally (pg. 37) - Provide understandable, culturally appropriate education that promotes dialogue, and is tailored to the needs and abilities of patients (pg. 37) and include communication strategies (pg. 80) - Provide evidence-based interventions according to patient plan of care (pg. 75) - Recommend exercise tailored to patient abilities and interests (pg. 75) | Clinicians should reassure patients that depression is a mental illness not a character defect (pg. 78) | N/A | Care should include:   - Shared decision making and power and respecting autonomy (pg. 37) - Respect for individuals' choices (pg. 40) - Creation of an individualized plan considering patient preferences and informed decision making supported (pg. 74) - Clinician personal opinions/beliefs should not influence patient therapy decision making (pg. 74) - Patient wishes should guide family/care partner involvement in education (pg. 78) | Clinicians should identify and address factors affecting adherence to treatment plan and arrange follow up care (pg. 74)  Plan of care should be collaborative and well communicated (pg. 74)  Several self-management strategies outlined (pg. 77-78) | N/A | Person and family-centred care mentioned as a guiding principle (pg. 31) defined as clinician:   - Knowing patient holistically - Honouring autonomy and decision making - Sharing power - Communicating verbally and non-verbally that they care and respect patient (pg. 37)   Educational content should be person and family centered (pg. 80) | N/A |
| US Preventive Services Task Force, 2016, United States [41] | National Academy of Medicine In Guidelines We Can Trust, and U.S. Preventive Services Task Force Procedure Manual | N/A | N/A | Discusses commonly used screening tests for assessing depression (pg. 382) | N/A | Treatment choices should be made through shared decision making (pg. 385) | Recommends collaborative care including (pg. 383):   - Self-management - Support - Care coordination - Ongoing support for medication adherence - Follow up assessments | N/A | Mentions that patient preferences and goals should be discussed at initial visit (pg. 383) | Women are at higher risk for depression (pg. 382)  Pregnant and postpartum women should be considered (pg. 384-5):   - Screening test accuracy and efficiency - Effectiveness of treatment - Potential harms of treatment and screening - Magnitude of net benefit |
| Cancer Care Ontario, 2015, Canada [36] | AGREE | Highlights the importance of empathetic communication and good patient-provider communication (pg. 12) | Family members should be involved in education and communication with clinician regarding prognosis and problems within support network (pg. 7) | Clinicians should destigmatize depression by reassuring patients that it is a serious issue needing treatment not a personal weakness or failure to cope (pg. 7) | N/A | Treatment options should be discussed in consideration of patient preferences and previous treatment experiences (pg. 7) | Collaborative care interventions: multiple providers coordinate in care for patient to enable self-management and hold weekly case review meetings (pg. 9) | N/A | Referred to patient preferences (pg. 7) and person-centred model for cancer care delivery (pg. 13) | N/A |
| Royal Australian and New Zealand College of Psychiatrists, 2015, Australia & New Zealand [33] | National Health and Medical Research Council | Therapeutic relationship should involve:   - Understanding the patient in the context of their unique circumstances (pg. 19) - Respect, support, warmth, empathy (pg. 21) - Maintenance during maintenance phase of treatment to keep patient engaged (pg. 61) | Patient should be characterized as an individual and contextual factors for why they have a mood disorder should be explored (pg. 18) | Patient may place importance on a therapeutic relationship which fosters respect, support, warmth and empathy (pg. 21) | A primary clinical coordinator should be involved when multiple clinicians are involved in care to mitigate patient and family hearing multiple views and opinions from care providers, lessen uncertainties about the best course of action and minimize stress on the patient (pg. 25) | Some authorities argue for collaborative treatment plans involving shared decision making (pg. 24). Patient preferences should be considered in decision about course of treatment (pg. 31) | Person should be engaged in the management of their condition (pg. 24) | N/A | Patient centric perspective described in relation to treatment of chronic illnesses such as mood disorders (pg. 24) | Menstruation and oral contraception use should be considered (pg. 20)  Being female increases risk of depression relapse (pg. 63) Treatment recommendations and special considerations for women with depression during pregnancy, post-partum and breastfeeding are discussed (pg. 122-124) |
| Toward Optimize Practice, 2015, Canada [29] | Institute of Medicine Standards for Developing Trustworthy Guidelines, AGREE | N/A | N/A | Recommends screening and recognizing depression and suicidality in patients with multiple sclerosis throughout document | N/A | Treatment should be chosen based on patients individual needs and circumstances (pg. 7) | Clinician should:   - Indicate patient responsibility to self-manage (pg. 3) - Check in on patient routinely (pg. 3) - Suggest CBT as a tool for developing self-management skills (pg. 10) | N/A | Mentions that treatment should be individualized and consider patient preferences and circumstances (pg. 7) | N/A |
| BC Reproductive Mental Health Program & Perinatal Services BC, 2014, Canada [28] | Not Reported | N/A | N/A | Discusses screening tools that clinicians can use to screen for depression in pregnant/postpartum women (pg. 19) | N/A | N/A | Self-management strategies for women with depression discussed (pg. 24) | N/A | N/A | Discusses barriers to women seeking help for mental disorders (pg. 15), and self-management strategies for women with depression (page 24) |
| BC Guideline, 2013, Canada [30] | Oxford Centre for Evidence-Based Medicine Levels of Evidence | N/A | Specific information that should be given by clinicians during pharmacotherapy management is outlined, paired with a note that clinicians should engage patients in an open discussion about the side effects of antidepressants (pg. 4) | Recommends the "two quick question" method of screening for depression in patients who present with depressive symptoms (pg. 1) | N/A | N/A | Clinicians should (pg. 3-4):   - Recommend self- and lifestyle-management - Provide education and self-management resources - Follow-up with patients monthly/bimonthly to monitor for treatment response and prevent relapse - Care teams should be coordinated via inter-professional communication | N/A | N/A | N/A |
| Canadian Task Force on Preventive Health Care, 2013, Canada [27] | GRADE | N/A | Majority of patients find screening for depression to be important (page 779). Where screening leads to a diagnosis, patients' preferences and culture should be considered in further care (pg. 779) | Recommends against screening for depression among high risk adults. Suggests being alert to the possibility of depression in patients with clinical clues such as insomnia, low mood, anhedonia, suicidal thoughts (pg. 779) | N/A | Treatment should be culturally sensitive and consider patient preferences in order to improve outcomes (pg. 779) | Suggested integrated staff-assisted systems to enable effective self-management and follow-up care, thus increasing treatment success (pg. 780) | N/A | Referred to patient preferences (pg. 779) | Majority of patients who viewed screening for depression as important were women undergoing perinatal care (pg. 779). Postpartum women are at higher risk for depression than the general population |
| Kaiser Permanente Care Management Institute, 2012 United States [39] | GRADE | N/A | N/A | Discusses various treatments which address depressive symptoms throughout document (ex. antidepressants and psychotherapy on pg. 5) | N/A | Choice of antidepressant should be based on patient and clinician preference (pg. 5). Treatment preferences should especially be considered among ethnic groups, as their preferences may be influenced by culture and ethnicity (pg. 9) | Clinician should initiate follow-up care especially during the maintenance and continuity phases (pg. 8-9). Self-management strategies discussed (pg. 10) | N/A | Patient-centered, quality care mentioned as an important part of clinical practice (pg. 1). Patient-centered model of care and communication proposed (pg. 8, footnote) | Addresses the use of antidepressants during pregnancy or while breastfeeding and mentions that clinicians should discuss the risks with women (pg. 11, 98) |
| Scottish Intercollegiate Guidelines Network, 2012, United Kingdom [42] | Healthcare Improvement Scotland (SIGN) 50: A guideline developers handbook, GRADE | N/A | Effects of psychotropic medications on pregnancy and the use of reliable contraception methods should be discussed (pg. 20). An education/information checklist was created for clinicians treating women with perinatal mood disorders. Notes that info given should consider women's individual circumstances (pg. 27) | Clinical questionnaires should be used to facilitate discussion of emotional issues (pg. 13) | N/A | Decision making should involve:   - Patient preferences for treatment (pg. 14) - Involvement of woman and family (pg. 19) - Individualized risk- benefit assessment (pg. 19) - Support women's individual decision about breast feeding   Clinicians should be aware that psychotropic medication is not usually a contraindication for breastfeeding (pg. 25) | Women at high risk for mental illness should have a management plan that outlines who their support team is (including care providers) and who to contact in an emergency (pg. 10) | N/A | Patient preferences mentioned in relation to treatment (pg. 14) | Discusses various factors that should be used to predict risk of antenatal depression (pg. 7):   - Life stress - Lack of social support - Domestic violence - Relationship factors |
| National Institute for Health and Care Excellence, 2011, United Kingdom [43] | National Institute for Health and Care Excellence Developing NICE Guidelines | N/A | Clinicians can detect emotional distress using (pg. 37):   - Communication skills - Eye contact - Good interview skills - Asking well-formulated - Focusing on more than just a symptom count   Clinicians information should (pg. 89, 134):   - Discuss services available - Consider patients previous knowledge - Be in a range of languages and formatting including verbal and non verbal communication - Available in a variety of settings - Engage with family where appropriate (pg. 228) | Discusses tools for assessment of depression such as the Beck Depression Inventory (pg. 38). Identifies strategies for detecting emotional distress (pg. 37) | Clinicians should discuss uncertainty associated with effectiveness of counselling/psychodynamic therapy in treating depression (pg. 170) | When deciding on the best treatment, patient preferences should be considered (pg. 175) | Clinicians should ensure that services are in place for coordination and continuity of care (pg. 89) and advise patients about support groups, rehabilitation programs and educational and employment services (pg. 223) | Clinicians should be respectful and sensitive to diverse culture, ethnicity, and religions and consider these factors when implementing treatment plans (pg. 90) | Described their intent as to create a patient-centred, evidence based guideline (pg. 9, 41) | Special considerations for antenatal and postnatal mental health assessments were mentioned (pg. 124). Pregnancy and breast feeding should be considered before using drug treatment (pg. 135). Support should be provided to pregnant women with subthreshold symptoms (pg. 177) |
| American Psychiatric Association, 2010, United States [38] | American Psychiatric Association Guideline Development Process | Patient-clinician relationship can be maintained via:   - Clinicians should address issues by collaborating with patient (pg. 16) - Establishing rapport with patient regardless of treatment chosen (pg. 23) - Open discussion about goals and framework of treatment (pg. 23) - Be aware of transference and countertransference issues in management of this relationship (pg. 23) - Create a trusting environment where patients feel comfortable opening up about emotions (pg. 23) | Education should be (pg. 16-17):   - Conveyed in a language the patient understands - Include family if patient consents - Address common misconceptions - Open discussions with patients regarding misconceptions about psychiatric treatment, prior treatment experiences (pg. 23) - About goals and treatment frameworks (pg. 23) | Psychiatrist should encourage patient to discuss fears or concerns about treatment/side effects (pg. 16). Clinicians should realize that patients may have feelings of embarrassment, shame, guilt, alienation and unworthiness, and provide empathy, reassurance, and foster a trusting relationship where patients can open up about their feelings (pg. 23) | N/A | Patients and clinicians should collaborate on decision making (pg. 15) Patient preferences and concerns about treatment options should be considered (pg. 15). Patient preferences and prior treatment experiences should be considered when making a choice (pg. 17) | Clinicians should:   - Consider patient's ability to care for themselves when deciding on treatment (pg. 16) - Help patients set goals appropriate to their functional ability and symptom severity (pg. 16) - Help ensure that care is coordinated and synchronized and follow up care is a priority (pg. 16, pg. 19) | N/A | Referred to patient preferences and needs (pg. 15, 23) | Special considerations for treatment of women are discussed in detail (pg. 20-21):   - Fluctuation of gonadal hormone levels results in the need for detailed assessment of mood changes across reproductive phases. - Drug-drug interactions between anti-depressants and oral contraception must be considered - Side effects of SSRIs during premenopausal period (ex. relief of hot flashes) - Pregnant women should be educated on the risks and benefits of various treatments regarding the mother and fetal health - Women who are breast-feeding have to consider that newborns may receive anti-depressants via milk - Doses may need to be adjusted due to pharmacokinetic changes during pregnancy - Alternative treatments considered for pregnant women with psychotic or catatonic features (ex. electro-convulsive therapy). - Women's roles as caretakers in their family should also be assessed (ex. if patient is taking care of an ill relative, spouse etc.) |

**Cardiovascular Disease**

| Developer | Title | Fostering Relationship | Exchanging Info | Responding to Emotions | Managing Uncertainty | Making Decisions | Enabling Self-Management | Other PCC Elements Mentioned | General/Any Mention | Women’s Health Data |
| --- | --- | --- | --- | --- | --- | --- | --- | --- | --- | --- |
| Heart Failure Society of America, 2017 United States [58] | Institute of Medicine Clinical Practice Guidelines We Can Trust, ACCF/AHA Task Force on Practice Guidelines Methodology Manual and Policies | N/A | Yes - Recommendations provided should be based on individual values and preferences (pg. 4) | N/A | N/A | Yes - Shared decision making between clinicians and patients enhances adherence to recommendations such as the ones in these guidelines and treatment adherence (pg. 4) Patient engagement in selecting treatment is important (pg. 4). Shared decision making should guide choice of antihypertensive agents (pg. 25) | N/A | N/A | Yes - mention of patient preferences and values (pg. 4) | N/A |
| Scottish Intercollegiate Guidelines Network, 2017, United Kingdom [61] | Healthcare Improvement Scotland (SIGN) 50: A guideline developers handbook, GRADE | Relationship between patient and case manager is likely to influence success of self management (pg. 8) | Many patients stop medications without discussion with their doctor, leading to discrepancies in information/care (pg. 20). | Psychological care strategies (ex. CBT) for depression and anxiety discussed as cardiovascular disease puts patients at higher risk for these conditions (pg. 15-18) | N/A | Individual assessment needs are better addressed via a case manager because it results in a personalized care plan involving shared decision making (pg. 8). | Regarding initial assessment, patients should undergo individual assessment leading to care plan and intervention specific to their needs (pg. 5). Patients should be offered individualized management interventions depending on their lifestyle (ex. Smoking cessation program) (pg. 5) Strategies for maintenance of patient engagement (ex goal setting, action planning etc.) are discussed (pg. 6). Support systems are also discussed (pg. 7). Relationship with care manager likely to effect self management, through individualized goal setting and other interventions (pg. 8) | N/A | Referred to patient-centred approach to cardiac rehabilitation in relation to equality of care regardless or smoker status/dietary factors (pg. 1) | Mentioned that women and other minority groups are underrepresented, and suggests specific cardiac rehabilitation programs for women (pg. 6). Also mentioned that female patients tend to exhibit more anxiety when their partners attend cardiac rehab programmes with them. (pg. 7) |
| Scottish Intercollegiate Guidelines Network, 2016, United Kingdom [60] | Healthcare Improvement Scotland (SIGN) 50: A guideline developers handbook, GRADE | Yes - Open communication and compliance with patients wishes and individualised care as well as attention to therapeutic detail are crucial for end of life care (page 36) Doctor-patient relationship important for enabling communication (pg. 38) | Yes - Strategies for improving education and communication were discussed and a checklist of information that patients and carers may find helpful was included (pg. 39) | Yes - Heart failure patients should be screened for depression, and CBT should be considered as treatment (pg. 6, 14) | Yes - Option to discuss the uncertainty associated with end of life care in cardiac heart failure should be available at all stages of care (pg. 35) | Yes - Patient goals should be considered when choosing treatment strategy (pg. 28) | Yes - Several lifestyle management strategies were discussed (pg. 15 - 17), and post discharge support/care/follow up was recommended (pg. 33). Suggests that self management programmes should be tailored to individual patient requirements and pay attention to those with low literacy (pg. 34) | N/A | Yes - referred to patient centred care for end of life care in this patient group (pg. 36) | N/A |
| European Society of Cardiology, 2013, Europe [62] | Not Reported | N/A | Patients frequently request information about how to resume sexual activity as this is a common concern, and information is often not readily available to patients. It is mentioned that clinicians often have the expectation that the patient doesn't want this information, and cultural or language barriers may also inhibit this discussion (pg. 3218). Clinicians should ascertain the need for sexual counselling as well as patient preferences based on timing of this information (pg. 3219). Suggests giving permission to patients to discuss this topic via sating "after a heart attack, patients and their partners may have concerns about whether it is safe to engage in sex. What concerns do you have" and providing limited/general information. If the patient asks specific/individualized questions, the clinician should be prepared to discuss (pg. 3221-3222 | Yes - Patients report anxiety, fear, insecurity concerns about returning to sexual activity after a coronary event, and physicians often cite embarrassment and fear of upsetting the patient as reasons why they don't discuss these issues with patients, patients also report embarrassment as a barrier to bringing up this topic with clinician (pg. 3218). Providers should discuss patients fears about having sex after a cardiac event as soon as possible and provide reassurance that the risk of myocardial infarction is low (pg. 3221). | N/A | N/A | N/A | Clinician traits such as cultural background, stereotypical views, overprotective judgements act as barriers to discussions about patient concerns regarding sexual activity after a coronary event (pg. 3218) | Refers to consideration of variation in patient individual needs and preferences (pg. 3219) and refers to patient-centred, reflective, negotiated forms of communication (pg. 3222) | It is unknown whether women and men have different needs and experiences related to cardiovascular disease and sexual counselling, there is considerably less research regarding female sexual dysfunction post cardiac event (pg. 3220) |
| International Council of Cardiovascular Prevention and Rehabilitation, 2013, International [64, 65] | GRADE | N/A | N/A | Assessments for depression should be conducted and treatment should be based on patient preference and availability (pg. 3-4) | N/A | N/A | Clinicians should offer patients recovering from a major coronary heart disease event programs of exercise, smoking cessation counselling, personalized education about health goals (pg. 2 - 3) | N/A | N/A | N/A |
| American Heart Association: Prevention of Cardiovascular Disease in Women, 2011, United States [57] | Not Reported | N/A | Health disparities often occur when patients are unable to communicate their symptoms due to language barrier or other factors (pg. 7). Cultural competence is importance for clinicians to meet the needs of diverse healthcare populations (pg. 7). A team approach should be take towards patient education, including clinician, family, and patient (pg. 8) | N/A | N/A | Yes | Clinicians should involve patient and family in short term achievable goals and frequent follow up visits to improve success and treatment adherence (pg. 9). Several self management suggestions are listed (pg. 10) | N/A | N/A | In addition to the document being about CVD in women, the authors also touch on the health disparities associated with black and Hispanic women, and mention that the root cause of this is clinician lack of understanding of health beliefs, cultural values and preferences and patients' inability to communicate symptoms in a language other than their own. (pg. 6-7). Also mentions that women have other factors influencing their treatment adherence such as caretaking responsibilities, sleep deprivation, fatigue, stress and lack of personal time (pg. 9) |
| American Heart Association: Secondary Prevention and Risk Reduction Therapy for Patients With Coronary and Other Atherosclerotic Vascular Diseased, 2011 United States [66] | Not Reported | N/A | Patients should be asked about tobacco use status and be advised to quit/willingness to quit should be assessed at every visit (pg. 2433). Patients should be advised to avoid exposure to second hand smoke at work, home, and public place (pg. 2433). | Patients should be screened for depression (pg. 2436) | N/A | N/A | Patients should be assisted via counselling and development of a plan for quitting smoking (ex. Smoking cessation program or pharmacotherapy), and counseled regarding blood pressure control via lifestyle factors, encouraged to do physical activity, and counselled to report and be evaluated for symptoms related to exercise, and encourage weight management/reduction through an appropriate balance of lifestyle (pg. 2433 -2434). Patients in cardiac rehabilitation should be referred to a comprehensive outpatient rehab program/home based cardiac rehabilitation program/ or comprehensive exercise-based outpatient cardiac rehabilitation program (pg. 2436) | N/A | N/A | Mentions that ethnic minorities, women and the elderly are underrepresented in trails (pg. 2438) |
| European Society of Cardiology, 2011, Europe [63] | Not Reported | N/A | Mentions that women with heart disease should be given individual pre-pregnancy counselling to enable a risk assessment (pg. 3157). Mothers should be informed about the symptoms of venous thromboembolism in pregnancy and warned that they should contact their doctor if these symptoms occur (pg. 3187) | N/A | N/A | N/A | N/A | N/A | N/A | N/A |
| National Institute for Health and Care Excellence, 2010, United Kingdom [59] | National Institute for Health and Care Excellence Developing NICE Guidelines | N/A | Good communication between patient and clinician, supported by evidence based written information, tailored to patient needs, and accessible to patients is essential. Information should be culturally appropriate. Good communication includes: listening to patient, respecting views and beliefs, giving patients information they ask for or need, in a way that they understand, provide most important info first, explain how each item will affect patients personally, present info in separate categories, make advice specific detailed and concrete, confirm understanding via questions, defining unfamiliar words, writing down key works and use of diagrams - a copy should be kept in medical file (pg. 26-27) | N/A | Issues about sudden death and living with uncertainty should be discussed/available opportunity to discuss at all stages of care (pg. 28) | People with chronic heart failure should be given the chance to make informed decisions about their care and treatment, as a partnership with their clinicians and families should be involved where appropriate (pg. 8) | Timing of discharge should consider patient wishes and level of support available in the community (pg. 11, 25). Management should be seen as shared between patient and clinician, clinician should recommend support groups (pg. 27). | N/A | Patient centred care described as considering patients' needs and preferences (pg. 8) | Mentions that women with heart failure at the reproductive age should discuss contraception and pregnancy with clinician. If pregnancy occur, specialist care should be sought (pg. 22) |
